# The Urgent Need for Management of Biological Samples and Data Accessibility in Latin America

**DOI:** 10.3389/fphar.2021.620043

**Published:** 2021-03-19

**Authors:** Rodrigo José Vargas, Oscar M. Cobar

**Affiliations:** ^1^ Escuela de Estudios de Postgrado, Facultad de Ciencias Médicas, Universidad de San Carlos de Guatemala, Guatemala City, Guatemala; ^2^ Universidad Galileo, Guatemala City, Guatemala; ^3^ Latin American Network for the Implementation and Validation of Pharmacogenomic Clinical Guidelines (RELIVAF-CYTED), Madrid, Spain; ^4^ Universidad del Istmo, Fraijanes, Guatemala

**Keywords:** biobank, Amerindian, COVID-19, mestizo, diversity

## Introduction

Elucidation of the human genome has increased understanding of human body responses to drug administration (Oprea et al., 2018; Nabirotchkin et al., 2020). Likewise, recent studies on human genetic diversity have shown that it is still necessary to delve into individual genetic differences, the adverse effects associated with drug metabolism, drug response variability with the diet, and even the human microbiome (Sharma et al., 2019). Access to biological samples in Latin America is essential to further understanding the presence of genes that may be associated with adverse effects and pharmacological interactions due to their diversity of populations and in anticipating the effects of new treatments.

The Coronavirus disease 2019 (COVID-19) pandemic has reinforced the urgent need to study the genetic differences among people with mild symptoms and those with complex responses to the disease (Ovsyannikova et al., 2020). During the pandemic, different drugs have been studied in the search for therapeutic alternatives to combat it, mainly due to the “Solidarity Trial” and “Repurposing Drugs” initiatives of the World Health Organization (OMS) (Harrison, 2020). Similarly, new drugs for actual and future diseases can be designed by using pharmacogenetic information. As an example, due to the diversity of therapeutic action mechanisms of a drug, it is necessary to in parallel study human cell susceptibility to the entry of SARS-CoV-2 in genetic world populations. Pharmacogenetics studies of their allele variants are essential. This approach is imperative in enabling national health systems to make informed decisions about the therapeutic strategies used, especially in countries with multiple ethnic groups.

Examples that illustrate the importance of understanding therapeutic effectiveness responses in target groups include the premature administration of hydroxychloroquine based on affect glycosylation of angiotensin converting enzyme-2, without information on genetic variability (Ferner and Aronson, 2020) and remdesivir administration (Beigel et al., 2020). Research on ethnic group pharmacogenetics in Latin America is still scarce (Sosa-Macías et al., 2016; Leitão et al., 2020), but there is evidence of pharmacogenetic differences (Suarez-Kurtz and Parra, 2018). For example, Mestizo populations are not considered a unique group for these types of studies, since miscegenation varies in each Latin American country (Botton et al., 2019).The COVID-19 pandemic has shown us that data and access to pharmacogenetic information are required for making clinical decisions in a prioritized and sometimes urgent ways.

The reservoirs of genetic material in biobanks in the United Kingdom (McInnes and Altman, 2020) and even in Africa (Matimba et al., 2008), allow researchers to quickly access information on the allelic distributions of important pharmacogenetic markers. However, in Latin America, biobanks for pharmacogenetic research purposes have had only slight development. The bioethical and legal aspects must be considered, and access to biomedical sample information is necessary in a region with high ethnical diversity and little-studied ancestral Amerindian populations.

## Biobanks

Biobanks can hold genetic data for a significant percentage of an entire population (Malsagova et al., 2020). In Estonia, e.g., genetic information on about 5% of the adult population can be found in a DNA bank (Reisberg et al., 2019). Additionally, biobanks contribute to reducing the cost of pharmacogenetic studies (Huttin and Liebman, 2013) avoiding the sampling of the population of interest in each study, also allowing a collaborative approach. In Europe, biobanks like one in Poland, have used different management models, preserving essential fundamental information for the benefit of public health (Sak et al., 2012). Moreover, biobanks are very useful for establishing pharmacogenetic relationships for research drug interactions with genes identified in a population (Muller et al., 2020; Rollinson et al., 2020).

## Bioethics and Legal Aspects

There are ethical dilemmas involved with asking a donor to provide unique informed consent. This has, however, been improved with the development of the model of dynamic consent (Steinsbekk et al., 2013). Ethics committees play a key role in the ethical debates concerning approval of donations to biobanks and access to the stored genetic material (Hansson, 2009). A legal framework is also required for the use of biobank samples (Helgesson et al., 2007). Recently, legal and ethical issues have focused on sample ownership and access, donor protection, and long-term storage of biological samples (Paskal et al., 2018; Facca et al., 2020).

## Conclusion

Due to high population diversity, Latin America faces the challenge of addressing genetic variability in studies to improve pharmacological responses to therapeutics for diseases. The creation of biobanks, their strengthening, and collaboration among them, would be a fundamental contribution to obtain pharmacogenetic information and efficient therapeutic responses in Latin America.
